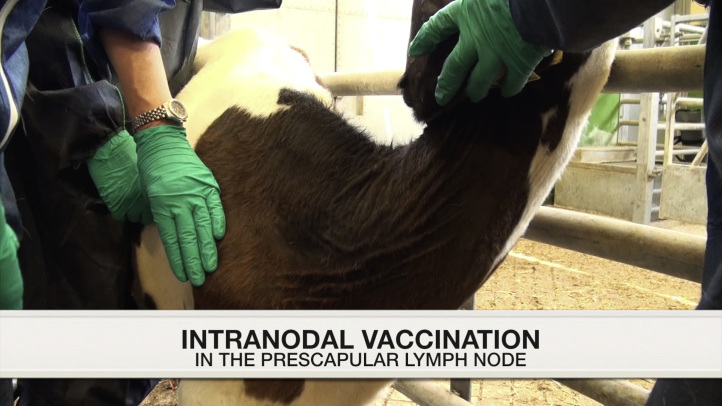# Development of a BCG challenge model for the testing of vaccine candidates against tuberculosis in cattle

**DOI:** 10.1016/j.vaccine.2014.08.009

**Published:** 2014-09-29

**Authors:** Bernardo Villarreal-Ramos, Stefan Berg, Laura Chamberlain, Helen McShane, R. Glyn Hewinson, Derek Clifford, Martin Vordermeier

**Affiliations:** a Bovine TB, AHVLA—Weybridge, New Haw, Addlestone, Surrey KT15 3NB, UK; b The Jenner Institute Old Road Campus Research Building Oxford University, Roosevelt Drive, Oxford OX3 7DQ, UK

**Keywords:** BCG, BCG challenge, Intranodal inoculation

## Abstract

Vaccination is being considered as part of a sustainable strategy for the control of bovine tuberculosis (BTB) in the UK. The live attenuated *Mycobacterium bovis* bacillus Calmette-Guerin (BCG) has been used experimentally to vaccinate cattle against BTB. However, BCG confers partial protection against BTB and therefore, there is a need to develop improved vaccines. BTB vaccine efficacy experiments require the use of biosafety level 3 facilities which are expensive to maintain, generally oversubscribed and represent a bottle neck for the testing of vaccine candidates. One indicator of the induction of protective responses would be the ability of the host's immune response to control/kill mycobacteria. In this work we have evaluated an intranodal BCG challenge for the selection of vaccine candidates at biosafety level 2 which are capable of inducing mycobactericidal responses. To our knowledge, this is the first such report. Whilst BCG only confers partial protection, it is still the standard against which other vaccines are judged. Therefore we tested the BCG intranodal challenge in BCG (Danish strain) vaccinated cattle and showed that vaccinated cattle had lower BCG cfu counts than naïve cattle at 14 and 21 days after intranodal challenge with BCG (Tokyo strain). This model could help prioritize competing TB vaccine candidates and exploration of primary and secondary immune responses to mycobacteria.

## Highlights

• Recovery of BCG from bovine tissues after vaccination.
• Potential novel routes of inoculation.
• Development of a BCG “challenge” model for the testing of vaccine candidates against bovine TB.

## 1 Introduction

*Mycobacterium bovis* belongs to the *Mycobacterium tuberculosis* complex of bacteria and is the main aetiologic agent of bovine tuberculosis (BTB) as well as being responsible for a proportion of cases of human tuberculosis (TB). Despite the application of the test and slaughter policy, the incidence of BTB in GB has increased steadily since the 1980s and this is thought to be due to the existence of a wildlife reservoir [1]. Hence, vaccination is being considered as an additional tool to contribute to the control of BTB [2]. The live attenuated strain *M. bovis* bacillus Calmette-Guerin (BCG) was derived from a case of bovine tuberculoid mastitis in the early 1900s and has been used as a vaccine against TB in humans since 1921 [3]. BCG has been used experimentally for vaccination of cattle against BTB since 1912, including in the UK in the first half of the 20th century [4], [5]. As in humans, BCG confers partial protection against BTB in cattle [6] and therefore, there is a need for better vaccines. It is possible to carry out vaccination and challenge experiments in cattle to determine whether a given vaccine or vaccination regimen confers protection against BTB. However, these experiments require the use of large animal biosafety level 3 (BSL3) facilities which are expensive to maintain and are often oversubscribed. Ideally, cheaper and faster gating criteria should be available to support the decision making process of whether a vaccine should be tested in cattle for protective efficacy in such vaccination and challenge experiments. This would considerably accelerate vaccine development.

Although BCG is attenuated, it is a live bacterium which replicates and survives in the host [3] and is normally handled in BSL2 facilities. If a vaccine is to be successful in conferring protection against challenge with virulent *M. bovis*, it should induce immune responses capable of controlling/killing mycobacteria and it is reasonable to propose that this could initially be demonstrated by an ability to induce a reduction in the number of BCG cfu. Recently, a human BCG challenge model for the testing of TB vaccine candidates has been described [7], [8]. We proposed that such a BCG challenge model in cattle, once developed, could serve as a gating criterion for this target species to screen vaccines before they are tested in expensive and facility-intense *M. bovis* challenge experiments. This paper describes the development of a cattle BCG challenge model.

## 2 Materials and methods

### 2.1 Cattle

Experimentation was carried out according to the UK Animal (Scientific Procedures) Act 1986. The study protocol was approved by the AHVLA Animal Use Ethics Committee (UK Home Office PCD number 70/6905).

Holstein-Friesian cattle of 4–6 months of age were sourced from farms known to be free of BTB.

### 2.2 Mycobacteria

The vaccine strain *M. bovis* BCG Danish 1331 was prepared as per manufacturer's instructions (SSI, Denmark). BCG Danish 1331 is currently the only BCG strain commercially available for vaccination.

The BCG challenge strain was BCG Tokyo (a kind gift from Dr. M Behr, McGill University, Canada), which was grown to mid log phase in 7H9 medium containing 0.05% Tween 80 (Sigma-Aldrich, Poole, United Kingdom) and ADC and stored frozen at −70 °C until further use. BCG Tokyo differs from BCG Danish 1331 at the RD2 and this difference would permit the distinction between the two strains in vaccination and challenge experiments. An aliquot was thawed and serial dilutions plated on 7H11 agar medium to determine bacterial titer. Frozen BCG Tokyo titer was determined to be at 1 × 10^7^ cfu/ml. Inoculum of BCG Tokyo used for challenge as well as selected colonies recovered from harvested lymph nodes were typed to confirm strain genotype. Samples were heat-inactivated at 80 °C and used as template in a PCR reaction using HotStarTaq Master Mix (Qiagen, United Kingdom) and three oligonucleotide primers (RD2_FW FlankFW, 5′-att gcg aac acg gga cgt cg-3′; RD2_FlankRev, 5′-gtt cgc cac aac ccg gaa cg-3′; RD2_InternalFW, 5′-gct cgt gtt tga cat cgg cg-3′) for large sequence polymorphism typing of the RD2 region [9]. PCR products of 196 bp and 319 bp defined the tested BCG isolates as RD2 intact (e.g. BCG Tokyo) and deleted (e.g. BCG SSI), respectively.

### 2.3 Vaccination and challenge experiments

Challenge experiment 1: For evaluation of optimal inoculation dosage, 16 animals were inoculated into the prescapular lymph node, which can be easily felt by palpation of the animal around the prescapular area; the lymph node was located and raised and the skin above the node was clipped and the node injected through the skin (please see Supplemental video). Animals were inoculated at day 0 with 10^7^ and 10^8^ cfu BCG Tokyo in 1 ml of 7H9 medium in the left and right prescapular nodes, respectively.

Challenge experiment 2: For vaccination and challenge, 48 animals were divided into four groups of 12 animals each; two of these groups were inoculated subcutaneously (s.c.) with 1-2 × 10^6^ BCG SSI in 0.5 ml Sauton's diluent in the left prescapular area. The other two groups were used as naïve controls; after eight weeks all 48 animals were inoculated in the right prescapular lymph node with between 1.8 × 10^8^ and 2.2 × 10^8^ cfu BCG Tokyo as indicated above.

### 2.4 Evaluation of immune responses

Immune responses were evaluated as production of interferon gamma (IFNγ) and IL-17 in whole blood as described elsewhere [10]. Briefly, peripheral blood was withdrawn from the jugular vein and placed in a tube containing sodium heparin (Leo laboratories) to a final concentration of 10,000 U/ml. Two hundred and twenty microliter of blood was incubated with 25 μl RPMI1640 medium alone (negative control [NC]) or with 25 μl *M. bovis* purified protein derivative (PPD-B) (10 μg/ml) (Prionics, Schlieren, Switzerland) and incubated at 37 °C in a 5% CO_2_ and 95% humidity atmosphere. After overnight incubation, blood was centrifuged at 300 × *g* for 10 min and plasma harvested and stored at −20 °C until use. Secretion of IFNγ was determined using the Bovigam™ assay (Prionics). Secretion of IL-17 was determined following the manufacturer's instructions (Kingfisher Biotec, MN, USA). Results are expressed as mean O.D. values ± standard error of the mean.

### 2.5 Determination of bacterial load in lymph nodes

After trimming, lymph nodes were submerged briefly in 70% ethanol prior to weighing and slicing for processing in a stomacher (Seward) for 2 min with 7 ml of PBS. Macerate was used to prepare serial dilutions for plating on modified 7H11 agar plates [11]. Results are presented as counts per ml.

### 2.6 Statistical analysis

Graph drawing and statistical analysis were carried out using GraphPad Prism v 5.02 (GraphPad Software, San Diego, CA) and GraphPad Instat v 3.06; for IFNγ secretion a Mann–Whitney ANOVA was carried out; for bacterial counts an unpaired *T*-test with Welch correction (significance indicated by ^*^ *p* < 0.05, ^**^ *p* < 0.01 or ^***^ *p* < 0.001 on the graphs). Statistical analysis for the spread of BCG to other lymph nodes was carried out with two sided contingency tables using Fischer exact test.

## 3 Results

### 3.1 Intranodal inoculation allows recovery of BCG

**Fig. 1:**
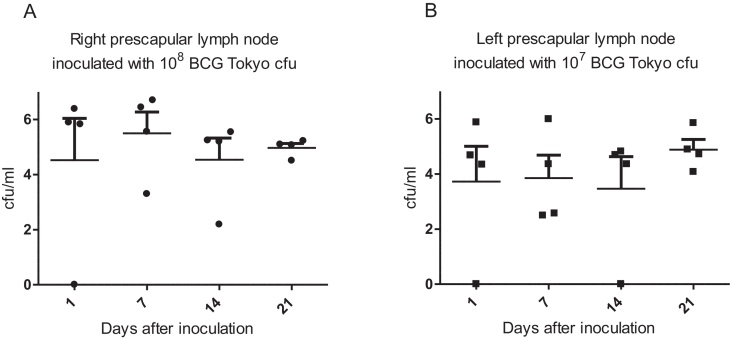
Recovery of BCG from prescapular lymph nodes. Cattle, four per time point, were inoculated with 10^8^ or 10^7^ BCG Tokyo cfu in the right (A) or left (B) prescapular lymph nodes. Lymph nodes were harvested at the time points indicated in the *x* axis and macerated as indicated in material and methods. Dilutions of lymph node macerates were plated in 7H11 agar plates for determining recovery of BCG. Bars represent the means and SEM.

To define the optimal dose and harvest time of the challenge organism, BCG Tokyo, 16 non-vaccinated cattle were inoculated intranodally with 10^7^ and 10^8^ cfu BCG Tokyo directly in the left and right prescapular lymph nodes, respectively. Lymph nodes, from four animals at each time point, were harvested at post-mortem 1, 7, 14 and 21 at days after inoculation. Fig. 1 shows the recovery of BCG from the prescapular lymph nodes at the different time points of harvest. Fig. 1A shows data following inoculation with 10^8^ cfu BCG Tokyo and Fig. 1B shows data following inoculation with 10^7^ cfu BCG Tokyo. Based on the observed data, we decided to undertake a proof of concept experiment in which cattle would be vaccinated with BCG Danish and challenged intanodally after 8 weeks with 10^8^ cfu BCG Tokyo and lymph nodes would be harvested at 2 and 3 weeks post-challenge (see below).

### 3.2 Immune responses to PPD-B induced by vaccination with BCG SSI and challenge with BCG Tokyo

**Fig. 2:**
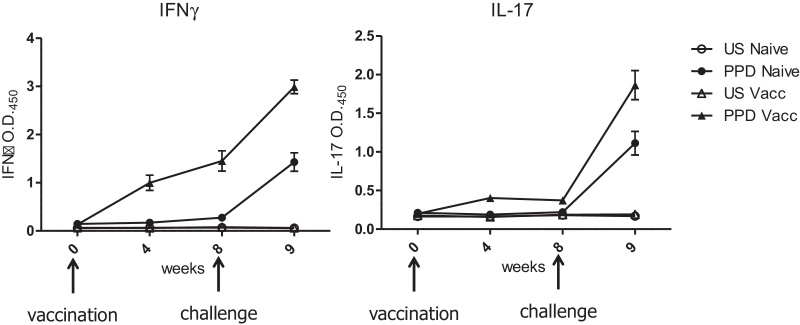
IFNγ secretion responses in whole blood following vaccination of cattle with BCG SSI. Cattle were vaccinated (triangles) or not (circles) with 10^6^ cfu BCG SSI subcutaneously and peripheral blood not stimulated (closed symbols) (US) or stimulated (open symbols) with PPD-B (PPD). Supernatants from blood were harvested and used in a Bovigam™ assay as indicated in materials and methods. Symbols represent the mean for each group and bars the SEM.

Based on the data from the experiment above, 48 cattle were divided into four groups of 12 animals each. Two groups were used as naïve controls and two groups were vaccinated subcutaneously (s.c.) in the left flank as described in materials and methods. To demonstrate vaccine take, the production of IFNγ and IL-17 after in vitro stimulation of whole blood with PPD-B was evaluated. Both, IFNγ (Fig. 2A) and Il-17 (Fig. 2B) were induced by vaccination with BCG. Responses to PPD-B were detectable in all vaccinated animals at week 4 and increased at week 8. No responses were detectable in naïve animals during this time period. IFNγ and IL-17 responses in naïve animals were induced by intranodal injection with BCG Tokyo, whilst previous BCG responses induced by BCG SSI in vaccinated animals were boosted at week 9.

### 3.3 Detection of BCG Tokyo in lymph nodes

**Fig. 3:**
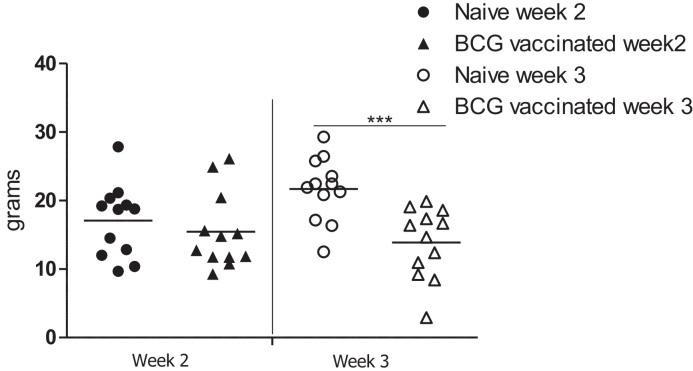
Inflammation, measured as increased weight of BCG-challenged lymph nodes, is greater in lymph nodes from naïve than from vaccinated animals. After challenge of naïve (circles) and BCG-vaccinated (triangles) cattle, prescapular lymph nodes were harvested at weeks 2 (closed symbols) and 3 (open symbols) and weighed. Results are presented for each individual animal and thick bars represent the mean for each group. ^***^p—0.0008 (unpaired *T*-test with Welch correction).

Eight weeks after vaccination, naïve and vaccinated animals were inoculated into the right prescapular lymph node with *c* 1 × 10^8^ cfu BCG Tokyo. To harvest lymph nodes, one group of BCG-vaccinates and one group of naïve control animals were killed at 2 weeks post-challenge and one group of BCG-vaccinates and one group of naïve control animals were killed at week 3 post-challenge. Prescapular, submandibular and popliteal lymph nodes were harvested at post-mortem. Fig. 3 shows the weights, as a measure of inflammation and cellular congestion, of the right prescapular lymph nodes; the nodes in which BCG Tokyo was injected. Whilst no significant difference in weight was detected in the lymph nodes from naïve and BCG-vaccinated cattle at week 2, corresponding comparison for week 3 showed that there was a statistically significant difference. At week 3 the lymph nodes from naïve animals were heavier (*ρ* = 0.0008); ranging from 12.51 g to 29.3 g with a median of 22.18 g while lymph nodes obtained from vaccinated animals ranged from 2.9 g to 19.89 g with a median of 15.52 g.

**Fig. 4:**
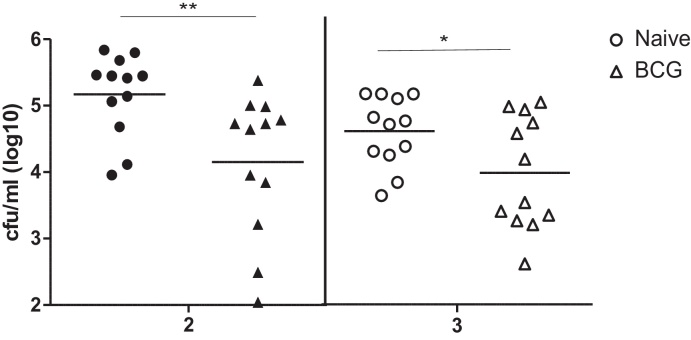
Bacterial counts, following BCG Tokyo challenge, are lower in vaccinated than in naïve animals. Eight weeks after BCG vaccination naïve (circles) and vaccinated animals (triangles) were injected intranodally with *c* 1 × 10^8^ cfu BCG Tokyo and lymph nodes were harvested at 2 (closed symbols) and 3 (open symbols) weeks. Results are presented for each individual animal and thick bars represent the mean for each group. ^*^p = 0.0107; ^*^p = 0.0439 (unpaired *T*-test with Welch correction).

Fig. 4 shows the bacterial counts from the right prescapular lymph nodes from naïve and BCG-vaccinated cattle at 2 and 3 weeks after inoculation. Lymph nodes from vaccinated animals showed statistically significantly lower bacterial counts at weeks 2 (*ρ* = 0.0107) and 3 (*ρ* = 0.0439) compared to lymph nodes from control animals after challenge. At week 2, the bacterial load in the right prescapular lymph nodes of naïve cattle ranged from 3.954 log_10_ cfu to 5.838 log_10_ cfu with a median of 5.431 log_10_ cfu; in the right prescapular lymph nodes from BCG-vaccinated cattle counts ranged from 2.041 log_10_ cfu to 5.38 log_10_ cfu with a median of 4.688 log_10_ cfu. At three weeks, the bacterial load in the right prescapular lymph node of naïve cattle ranged from 3.587 log_10_ cfu to 5.068 log_10_ cfu with a median of 4.648 log_10_ cfu; in the right prescapular lymph nodes from BCG-vaccinated cattle counts ranged from 2.591 log_10_ cfu to 4.944 log_10_ cfu with a median of 3.8 log_10_ cfu. The number of BCG cfu recovered from naïve animals at week 2 was higher than the cfu recovered at week 3; this difference was statistically significant (*ρ* = 0.0109). On the other hand, no difference was found in BCG cfu recovered at week 2 compared to week 3 in BCG vaccinated animals.

It was of interest to determine the distribution of the bacteria following challenge with BCG-Tokyo. To that effect, as well as evaluating bacterial counts in the right prescapular lymph nodes, counts were also evaluated in left prescapular lymph nodes and in left and right submandibular and popliteal lymph nodes. Table 1 shows the proportion of animals presenting bacterial counts in the different lymph nodes according to time and treatment. The data indicate that the dissemination of BCG Tokyo was greater in naïve control animals compared to animals that had been vaccinated with BCG at week 0. The differences at both 2 and 3 weeks were statistically significant (*ρ* = 0.0017 and *ρ* = 0.0005, respectively).

**Table 1:** Bacterial counts in lymph nodes to which mycobacteria could have spread. Data shown are number of animals in which at least one cfu was counted over the total number of animals inoculated with BCG Tokyo. Notice that the number of animals with bacterial counts is greater in the non-vaccinated than in the BCG vaccinates.

Lymph node	Week 2		Week 3	
	Non-vaccinated	BCG	Non-vaccinated	BCG
Left prescapular	8/12	3/12	6/12	1/12
Left submandibular	1/12	0/12	1/12	0/12
Right submandibular	2/12	0/12	2/12	0/12
Left popliteal	4/12	1/12	4/12	1/12
Right popliteal	3/12	0/12	3/12	0/12

## 4 Discussion

Vaccination and challenge experiments are a necessity for the development of vaccines against bovine TB. However, these experiments involve the use of large animal BSL3 facilities. Whilst necessary, due to their nature, these facilities are expensive to run and limited in number and therefore represent a bottle neck for the testing of vaccine candidates. Development of a model in the target species, cattle, for prioritizing vaccines under lower containment conditions would save money as BSL2 facilities are cheaper to run than BSL3 facilities. Being an attenuated strain of *M. bovis* it would be expected that cattle would at some stage control BCG and therefore the BCG challenge experiments would be shorter than standard virulent *M. bovis* challenge experiments. Further, by reducing the need for BSL3 experimentation, vaccine development programmes could be significantly accelerated.

An initial step for the development of such a model requires the establishment of a baseline for the consistent and reliable recovery of mycobacteria from bovine tissues. Previous attempts in this laboratory to recover BCG from cattle following s.c. challenge proved inconsistent. It is thought that following s.c. inoculation mycobacteria would migrate to the lymph node draining the site of inoculation; however, after inoculation, mycobacteria could disperse within the subcutaneous area and it is possible that mycobacteria could migrate to more than one node. By using intranodal inoculation, we have reduced the possibilities of mycobacteria dispersing within the subcutaneous areas and migrating to nodes other than the lymph node injected. To our knowledge, the experiment described in Fig. 1 is the first time in which a time curve, albeit partial to day 21, on the recovery of BCG from cattle has been reported. Thus, this is the first report for the relatively consistent recovery of BCG from cattle in quantifiable numbers.

This protocol was then used to determine whether prior vaccination using BCG SSI would affect the recovery of BCG after challenge compared to naïve animals in a manner similar to a standard efficacy vaccine test where virulent *M. bovis* is used for the challenge phase. Given the volume of literature and our previous experience, we decided to use BCG SSI as the test vaccine in these proof-of-principle experiments. We also decided to harvest lymph nodes after 2 and 3 weeks as we reasoned that this would be sufficient time for immune responses induced by previous vaccination to have an impact on the control of the BCG challenge and would maximise our ability to detect differences between vaccinated and non-vaccinated animals. On a group basis, prior BCG vaccination did reduce the number of mycobacteria recovered from vaccinated animals compared to non-vaccinated animals. However, from Fig. 4, it is clear that there was animal to animal variation in both vaccinated and naïve animals following inoculation with BCG Tokyo. It is also clear that not all BCG-vaccinated animals were protected to the same extent. It is possible to divide the animals into protected and not-protected by considering all BCG vaccinates with cfu counts lower than the animal presenting the lowest cfu counts in the non-vaccinated group as protected; all other BCG vaccinates could be considered as not protected. Using this criterion, 4/12 animals would have been protected by BCG vaccination after 2 weeks; at 3 weeks, 6/12 animals would have been protected. This outcome therefore parallels the outcome of vaccinated animals after challenge with *M. bovis*, with a proportion of animals presenting with pathology not indistinct from naïve control animals, and another proportion of animals presenting without or with significantly reduced pathology compared to naïve cattle [12], [13].

It is of interest that intranodal inoculation of naive cattle with BCG induced immune responses to PPD-B as early as one week after injection (week 9 for previously non-vaccinated animals). As observed in the BCG vaccinated group, IFNγ and IL-17 responses can be detected at between 2 and 4 weeks after vaccination [10], [14], [15]. It is not clear whether this phenomenon was due to the higher dose used during challenge or to the intranodal route of inoculation or that BCG Tokyo for challenge was derived from frozen logarithmic growth phase liquid stocks, whilst for vaccination lyophilised BCG SSI was resuspended in Sauton's medium. Intranodal inoculation has been reported to be more immunogenic than the intradermal or intravenous routes of immunisation [16], [17] and it is possible that this route of inoculation may induce stronger immune responses than those normally induced by BCG which may translate into greater protection against *M. bovis*. Future experiments will be necessary to test this hypothesis.

Whilst it was not the purpose of this study to establish the extent of dissemination of BCG in cattle, these experiments provide evidence that BCG spreads to organs other than those directly inoculated. However, it is important to state that these results cannot be correlated to what would happen following subcutaneous vaccination due to the following reasons: the strain used for challenge was BCG Tokyo from frozen mid-log liquid cultures whilst BCG SSI, the strain used for vaccination, is genetically different and was used as a lyophilised suspension. The dose used for vaccination was 100 fold lower than the dose used for challenge and the vaccine was administered s.c. whilst the challenge was given intranodally. It is also worth pointing out that, after challenge, BCG Tokyo was more widely distributed in non-vaccinated cattle than in vaccinated cattle. The bacteria obtained from lymph nodes other than the right prescapular lymph node, the site of injection, were confirmed by genetic typing to be BCG Tokyo and not BCG SSI (results not shown). Thus, we did not detect BCG SSI in the lymph nodes examined in these experiments at 10 (week 2 after challenge) and 11 (week 3 after challenge) weeks after s.c. inoculation.

In conclusion, this target species model can be used as a gating system for vaccine candidates prior to further testing in BSL 3 facilities using virulent *M. bovis* challenge. This model could also be used to further explore the bovine primary and secondary elements of an immune response against mycobacteria in order to determine which factors are important in the control and/or killing of mycobacteria.

## Funding

This work was supported by funding from the Department for International Development, U.K. and the Bill and Melinda Gates Foundation.

HMcS, RGH and HMV are Jenner Investigators.

## Conflict of interest statement

None.

## Figures and Tables

**Figure:**