# Supplementary figures and images for: Development of a BCG challenge model for the testing of vaccine candidates against tuberculosis in cattle
Source: Vaccine. 2014 Sep 29;32(43):5645–9. doi: 10.1016/j.vaccine.2014.08.009 (PMC5441994; doi:10.1016/j.vaccine.2014.08.009)

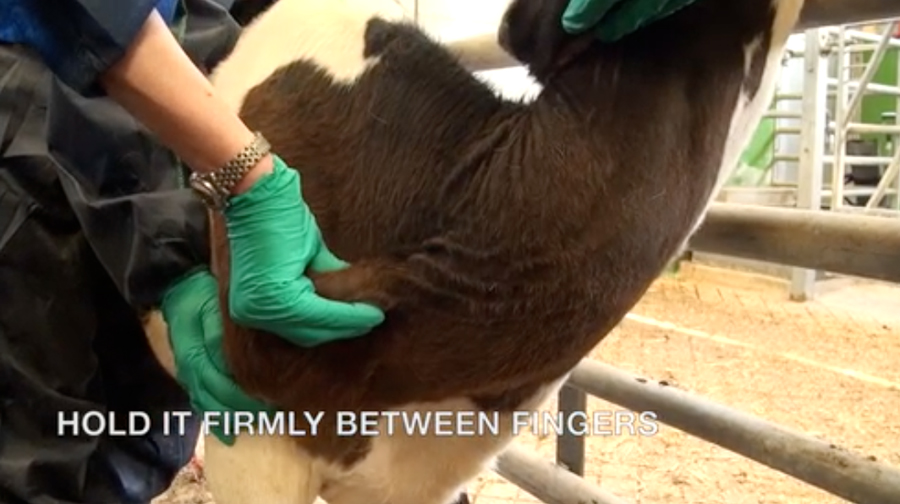

Supplement: Supplementary file 1 [file mmc1.jpg]
